# Role of Tachykinin 1 and 4 Gene-Derived Neuropeptides and the Neurokinin 1 Receptor in Adjuvant-Induced Chronic Arthritis of the Mouse

**DOI:** 10.1371/journal.pone.0061684

**Published:** 2013-04-23

**Authors:** Éva Borbély, Zsófia Hajna, Katalin Sándor, László Kereskai, István Tóth, Erika Pintér, Péter Nagy, János Szolcsányi, John Quinn, Andreas Zimmer, James Stewart, Christopher Paige, Alexandra Berger, Zsuzsanna Helyes

**Affiliations:** 1 Department of Pharmacology and Pharmacotherapy, Faculty of Medicine, University of Pécs, Pécs, Hungary; 2 János Szentágothai Research Center, University of Pécs, Pécs, Hungary; 3 Department of Pathology, Faculty of Medicine, University of Pécs, Pécs, Hungary; 4 Department of Molecular and Clinical Pharmacology, Institute of Translational Medicine Liverpool University, Liverpool, United Kingdom; 5 Laboratory of Molecular Neurobiology, Department of Psychiatry, University of Bonn, Bonn, Germany; 6 School of Infection and Host Defense, University of Liverpool, Liverpool, United Kingdom; 7 Ontario Cancer Institute, University Health Network, Toronto, Canada; 8 Department of Immunology, University of Toronto, Toronto, Canada; University Hospital Jena, Germany

## Abstract

**Objective:**

Substance P, encoded by the Tac1 gene, is involved in neurogenic inflammation and hyperalgesia via neurokinin 1 (NK1) receptor activation. Its non-neuronal counterpart, hemokinin-1, which is derived from the Tac4 gene, is also a potent NK1 agonist. Although hemokinin-1 has been described as a tachykinin of distinct origin and function compared to SP, its role in inflammatory and pain processes has not yet been elucidated in such detail. In this study, we analysed the involvement of tachykinins derived from the Tac1 and Tac4 genes, as well as the NK1 receptor in chronic arthritis of the mouse.

**Methods:**

Complete Freund’s Adjuvant was injected intraplantarly and into the tail of Tac1^−/−^, Tac4^−/−^, Tacr1^−/−^ (NK1 receptor deficient) and Tac1^−/−/^Tac4^−/−^ mice. Paw volume was measured by plethysmometry and mechanosensitivity using dynamic plantar aesthesiometry over a time period of 21 days. Semiquantitative histopathological scoring and ELISA measurement of IL-1β concentrations of the tibiotarsal joints were performed.

**Results:**

Mechanical hyperalgesia was significantly reduced from day 11 in Tac4^−/−^ and Tacr1^−/−^ animals, while paw swelling was not altered in any strain. Inflammatory histopathological alterations (synovial swelling, leukocyte infiltration, cartilage destruction, bone damage) and IL-1β concentration in the joint homogenates were significantly smaller in Tac4^−/−^ and Tac1^−/−/^Tac4^−/−^ mice.

**Conclusions:**

Hemokinin-1, but not substance P increases inflammation and hyperalgesia in the late phase of adjuvant-induced arthritis. While NK1 receptors mediate its antihyperalgesic actions, the involvement of another receptor in histopathological changes and IL-1β production is suggested.

## Introduction

Rheumatoid arthritis (RA) is a progressive, systemic auto-immune disease. Among adult Western white populations its prevalence is approximately 1% [1]. Pain and inflammation are the initial symptoms, followed by various degrees of joint destruction. In RA, pain is generally perceived to arise directly from inflammatory processes, as well as from the activation of central and peripheral neuronal mechanisms [2], [3]. The peripheral sensory nervous system with special emphasis on the capsaicin-sensitive peptidergic terminals densely innervating the joints, participates in inflammatory and pain processes. Proinflammatory sensory neuropeptides, such as tachykinins and calcitonin-gene related peptide (CGRP) in the target organs induce vasodilatation and recruitment of inflammatory cells to sites of inflammation [4], [5]. Increased sensory neuropeptide levels have been demonstrated in the serum and synovial fluid taken from RA patients [6], [7] and arthritic animals [8]. More recently Tac1 has been demonstrated to be transiently expressed in non- neuronal cells in response to challenge including chondrocytes and epithelical cells. In several models such expression is implicated in the initiation and progression of the inflammatory process [9], [10], [11].

Mammalian tachykinins are 10–12 amino acid peptides sharing the hydrophobic C-terminal region FXGLM-NH_2_. Substance P (SP) and neurokinin A (NKA), encoded by the preprotachykinin A (Tac1) gene, are expressed predominantly in capsaicin-sensitive primary sensory neurones of the dorsal root ganglia, although transient expression in response to challenge is seen in a variety of non-neuronal cells. Neurokinin B (NKB), derived from the preprotachykinin B (Tac3) gene, is found predominantly in the central nervous system. The newest member of the tachykinin gene family is the preprotachykinin C (Tac4) gene discovered in 2000 [12]. Tac4 encodes hemokinin-1 (HK-1) in mice and its equivalent peptides, endokinins, in human predominantly in immune cells, but also in various brain regions [13].

Three G-protein-coupled mammalian receptors have been identified, to which tachykinins have different affinities [4]. SP binds predominantly to the NK1 tachykinin receptor localized mainly on neuronal, endothelial and immune cells. NKA shows the greatest affinity to NK2 receptors, while NKB to NK3 receptors in the brain. HK-1 is very similar to SP regarding its structure and pharmacology, it has similar receptor binding and preference for NK1 [13], [14], [15], [16], [17]. However, some data on the basis of different actions compared to SP, strongly suggest that there might be a specific HK-1 receptor [18].

Tachykinins are conserved in mammalian species, and they are involved in several biological actions, such as smooth muscle contraction, vasodilation, pain transmission, inflammation, haematopoiesis, activation of the immune and endocrine system, and emotional behaviour [19], [20], [21]. In contrast to other tachykinins, HK-1 is mainly expressed in non-neuronal tissues. Tac4 expression levels are significantly lower in most neuronal, but higher in peripheral tissues compared to Tac1 [13]. Since Tac4 mRNA expression has been detected in a variety of immune cells, such as T and B lymphocytes, macrophages and dendritic cells, [12], [22], [23] HK-1 may have an important role in immune regulation.

Tachykinin research was florishing 15–20 years ago, and NK1 receptor antagonists were suggested as potential anti-inflammatory and analgesic agents according to their efficacy in animal experiments. However, most clinical trials did not reflect that promise [24], [25]. This might have been due to species differences in biology or pharmacodynamics of specific antagonists, but also to the fact that these agents were designed to block SP binding. The recent discovery of Tac4-derived tachykinins and evidence on the transient expression of Tac1 in non-neuronal cells revived the tachykinin research field [10]. Our present study represents an integrative analysis of the role and complexity of the tachykinin system in a murine model of chronic arthritis evoked by the administration of complete Freund’s adjuvant using genetically manipulated tachykinin and receptor knockout mouse strains.

## Methods

### Ethics Statement

All experimental procedures were carried out according to the 1998/XXVIII Act of the Hungarian Parliament on Animal Protection and Consideration Decree of Scientific Procedures of Animal Experiments (243/1988) and complied with the recommendations of the International Association for the Study of Pain and the Helsinki Declaration. The studies were approved by the Ethics Committee on Animal Research of University of Pécs according to the Ethical Codex of Animal Experiments and licence was given (licence No.: BA 02/2000–2/2012).

### Experimental Animals

Experiments were performed on male NK1 receptor (Tacr1^−/−^), Tac1^−/−^, Tac4^−/−^ and Tac1^−/−/^Tac4^−/−^ gene-deficient mice backcrossed for 8–10 generations to C57Bl/6 mice. C57Bl/6 mice were used as wildtype (WT) controls and the original breeding pairs were purchased from Charles-River Ltd. (Hungary). Tac1^−/−^ and Tacr1^−/−^ mice were generated at the University of Liverpool as previously described [26], [27], [28], [29]. Tac4^−/−^ and Tac1^−/−/^Tac4^−/−^ mice were obtained from Berger et al. [30], [31]. The animals were bred and kept in the Laboratory Animal House of the Department of Pharmacology and Pharmacotherapy of the University of Pécs at 24–25°C, provided with standard mouse chow and water ad libitum and maintained under a 12-h light-dark cycle.

### Induction of Arthritis

We have previously adapted the adjuvant-induced arthritis originally developed in Lewis rats [29] to mice to create a suitable experimental model for the examination of long-term joint inflammation in genetically manipulated animals [32]. Chronic arthritis of mice weighing 20–22 g (C57Bl/6 n = 24, Tac1^−/−^ n = 22, Tac4^−/−^ n = 18, Tacr1^−/−^ n = 19, Tac1^−/−/^Tac4^−/−^ n = 9; since there were 4 different gene-deleted strains and the study was performed in 5 experimental series in order to be able to precisely carry out all measurements, wildtypes were investigated in each series to minimize potential bias and differences induced by environmental changes.) was induced by intraplantar injection of 50 µl of Complete Freund’s Adjuvant (CFA, killed Mycobacteria suspended in paraffin oil, 1 mg/ml; Sigma, St. Louis, MO) into the right hind paw and s.c. into the root of the tail. An additional s.c. injection was given on the following day into the tail in order to potentiate the systemic effects and to make our model more similar to the human disease, as described in our earlier studies [32], [34].

### Measurement of Touch Sensitivity of the Paw

Touch sensitivity of the plantar surface of the paw was determined by dynamic plantar aesthesiometry (Ugo Basile 37400, Comerio, Italy) before and 4, 6, 8, 11, 13, 15, 18, 20 and 21 days after CFA administration. This device is a modified, electronic von Frey technique, which is used to assess mechanonociception. Mechanical hyperalgesia was expressed as % of control mechanonociceptive threshold compared to the initial values [29], [32], [33].

### Measurement of the Paw Volume

Paw volume was measured by plethysmometry (Ugo Basile Plethysmometer 7140, Comerio, Italy) [29], [32]. Volumes were measured prior to CFA-injection, and 4, 6, 8, 11, 13, 15, 18, 20 and 21 days after CFA administration. Oedema was expressed in percentage compared to the initial values [34].

### Histological Processing and Assessment of Joint Inflammation

Mice were anaesthetized with ketamine (100 mg/kg, i.p., Richter Gedeon Plc., Hungary) and xylazine (10 mg/kg, i.m., Lavet Ltd., Hungary), then sacrificed by cervical dislocation on day 21 after CFA administration and the paws were excised. After formaldehyde fixation, decalcification and dehydration the samples were embedded in paraffin, sectioned (5 µm) and stained with hematoxylin and eosin [29], [32].

Arthritic changes were scored by an observer blinded from the treatment the animals received using a grading scale of 0 to 3 according to the 1) proportion of the areolar tissue, infiltration by mononuclear cells, synovial lining cell hyperplasia, 2) the number of leukocytes observed in the synovial tissue, 3) cartilage destruction and 4) bone erosion. The score values given for these four different histopathological features were added to generate a composite arthritis score ranging between 0 and 12 [29], [32].

### Determination of IL-1β Concentrations in Tissue Homogenates

Excised paws were frozen in liquid nitrogen and kept at −80°C until further processing. Samples were homogenized in 1 ml buffer containing 990 µl RPMI 1640 medium and 10 µl PMSF (phenyl-methyl-sulphonyl-fluoride) protease inhibitor for 2 min at 21,000 rpm with Miccra D-9 Digitronic device (Art-moderne Laborteknik, Germany), centrifuged for 10 min at 5°C at 12,500 rpm, and the supernatants were stored at −20°C. The concentrations of the inflammatory cytokine IL-1β were measured by ELISA using IL-1β OptEIA set (BD Biosciences, USA).

### Statistical Analysis

All data were carefully tested for normal distribution (GraphPad Prism). Since hyperalgesia and oedema values followed normal distribution, they were evaluated by repeated measures two-way analysis of variance (ANOVA) followed by Bonferroni’s modified t-test in order to be able to compare the results at distinct timepoints. Since cytokine concentrations and semiquantitative composite arthritis scores were not normally distributed, they were analysed by the non-parametric Kruskal-Wallis test followed by Dunn’s post test to evaluate the differences between gene-deleted and WT mice. In all cases P<0.05 was considered to be significant, which are indicated in the graphs, where applicable.

## Results

### Role of Tachykinins in Adjuvant-induced Inflammatory Mechanical Hyperalgesia

**Figure 1:**
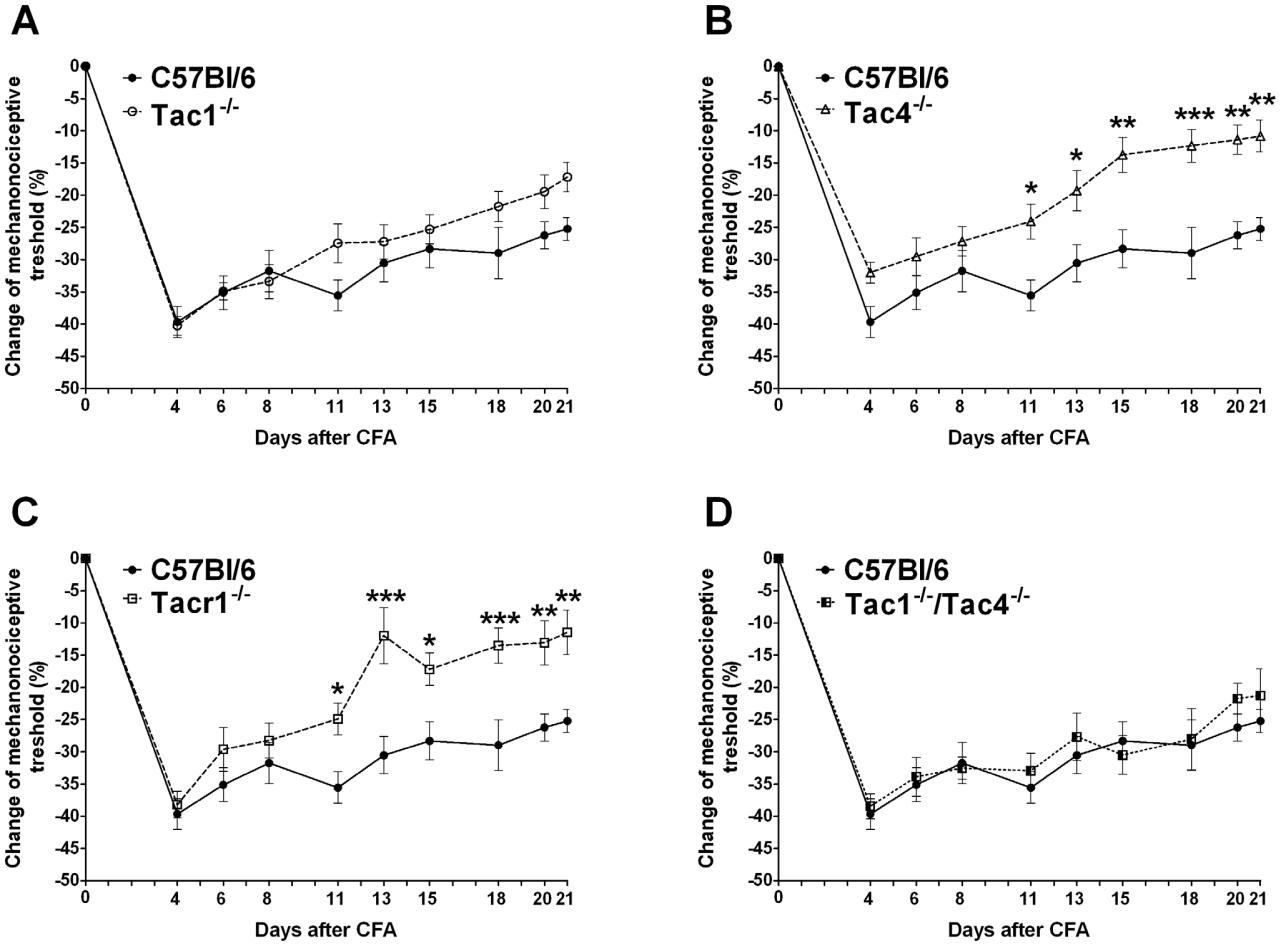
Adjuvant-induced mechanical hyperalgesia throughout the 21-day experimental period. Each data point represents the mean ± SEM of the percentage decrease of the mechanonociceptive threshold of (**A**) Tac1^−/−^, (**B**) Tac4^−/−^, (**C**) Tacr1^−/−^, (**D**) Tac1^−/−/^Tac4^−/−^ mice compared to the initial control values (n = 9–24 mice per group; *p<0.05, **p<0.01, ***p<0.001 vs. C57Bl/6; two-way ANOVA followed by Bonferroni’s modified t-test).

In WT mice an approximately 40% decrease of the mechanonociceptive threshold developed 4 days after adjuvant injection, which gradually decreased to 20% by the end of the study (Figure 1). Significantly reduced mechanical hyperalgesia was observed in the Tac4 and Tacr1 gene-deleted groups starting on day 11 of the experiment (Figures 1B, C). In contrast, no significant difference in pain thresholds was detected in either Tac1^−/−^ or Tac1^−/−/^Tac4^−/−^ mice (Figures 1A, D).

### Tachykinins are not Involved in Adjuvant-induced Oedema

**Figure 2:**
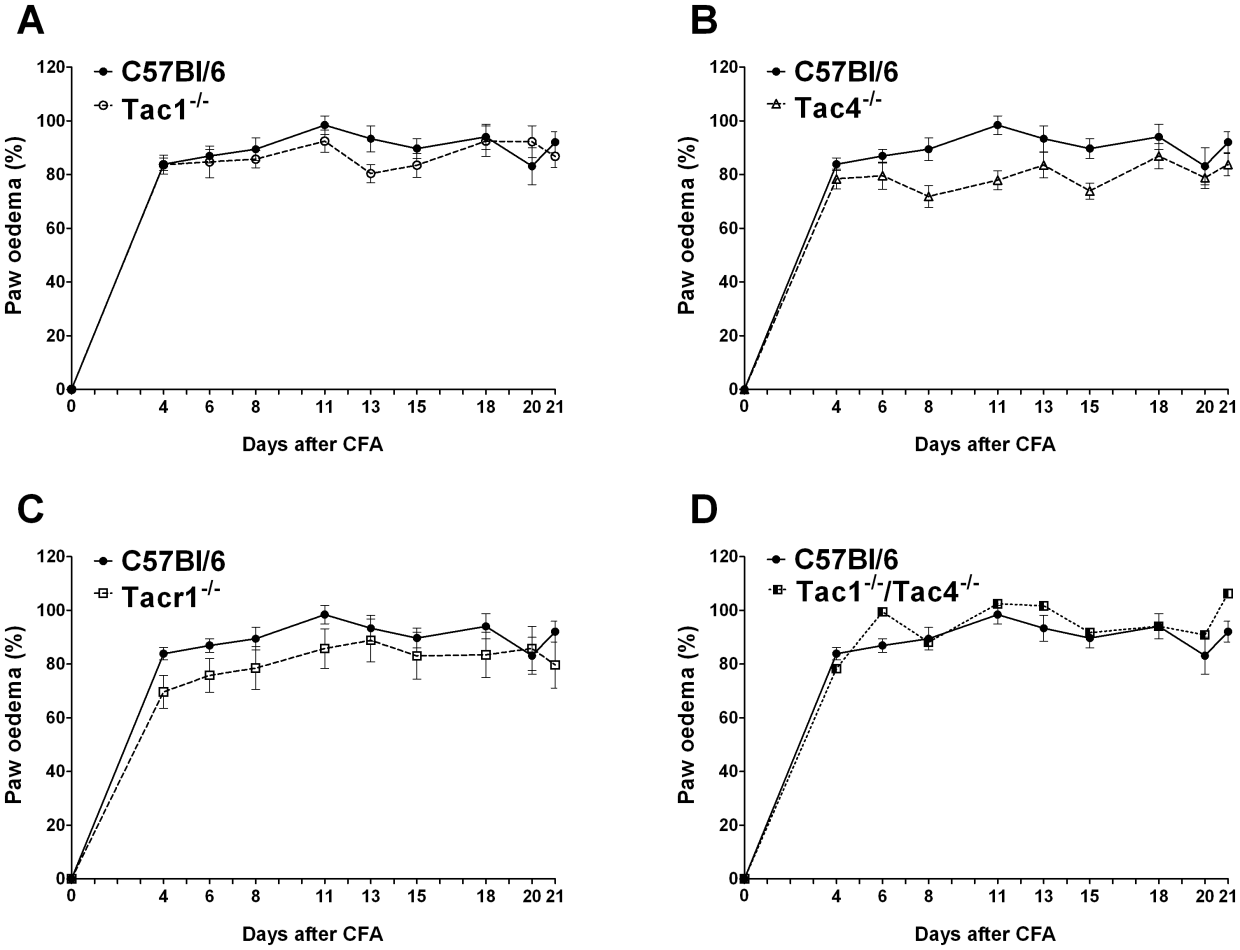
Adjuvant-induced oedema throughout the 21-day experimental period. Each data point represents the mean ± SEM of the percentage increase of the paw volume of (**A**) Tac1^−/−^, (**B**) Tac4^−/−^, (**C**) Tacr1^−/−^, (**D**) Tac1^−/−/^Tac4^−/−^ mice compared to the initial control values (n = 9–24 mice per group, two-way ANOVA followed by Bonferroni’s modified t-test).

In control animals, the volume of the CFA-injected paws increased to about 90% within 4 days post adjuvant injection, reaching a maximal swelling of approximately 98% 11 days after the induction of inflammation. No significant differences were observed in any knockout strains compared to controls (Figure 2).

### Role of Tachykinins in Adjuvant-induced Arthritic Histopathological Alterations

**Figure 3:**
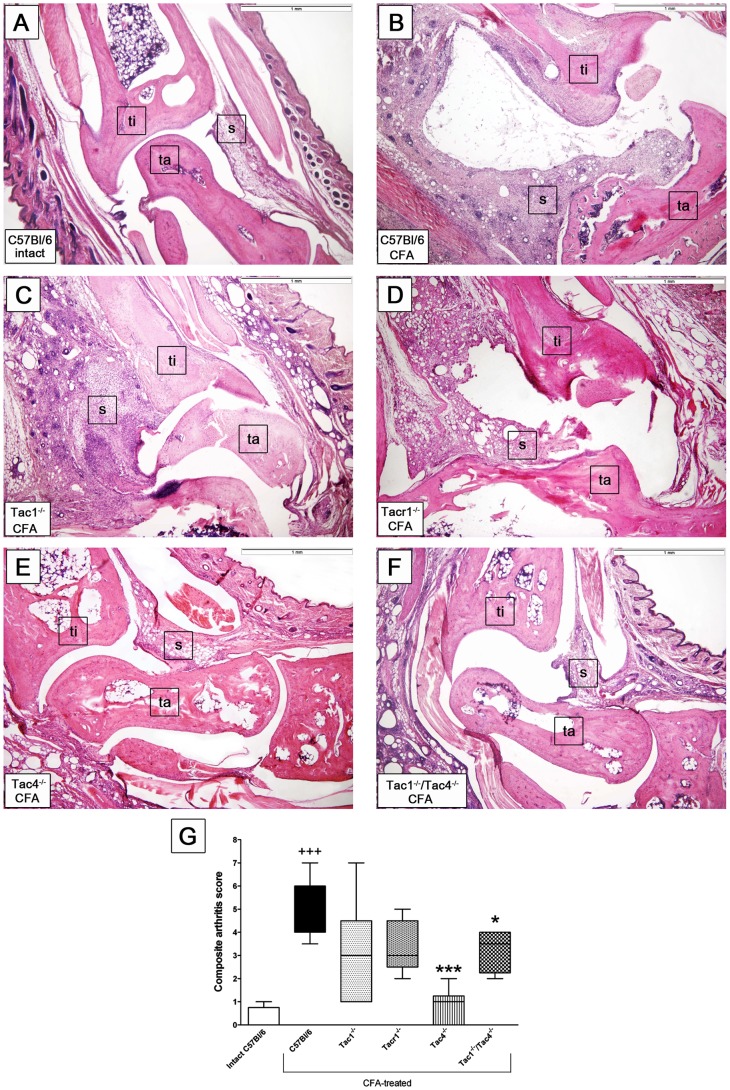
Histopathological changes of the paws on day 21. Panel **A** shows a representative histopathological picture of an intact tibiotarsal joint and panel **B** demonstrates the joint structure of an adjuvant-treated C57Bl/6 wildtype mouse with remarkable synovial swelling, leukocyte infiltration, cartilage damage, and bone destruction. The lower panes demonstrate the joint structures of adjuvant-treated (**C**) Tac1^−/−^, (**D**) Tacr1^−/−^, (**E**) Tac4^−/−^, and (**F**) Tac1^−/−/^Tac4^−/−^ mice, decreased inflammatory parameters can be observed in the latter two groups. Hematoxylin-eosin staining, 40x magnification (ti: tibia, ta: tarsus, s: synovium). (**G**) Semiquantitative histopathological scoring on the basis of inflammatory cell accumulation, synovial enlargement, cartilage destruction and bone erosion. Box plots represent the composite scores (n = 4–12 mice per group,^+++^p<0.001 vs. intact C57Bl/6, *p<0.05, ***p<0.001 vs. C57Bl/6 CFA-treated, Kruskal-Wallis followed by Dunn’s post test).

There was no difference between the intact joint structures of C57Bl/6 WT (Figure 3A) and any gene-deleted mice (data not shown). Meanwhile, the right tibiotarsal joints of adjuvant-injected WT mice were damaged by expanding synovial pannus. Widening of the synovial cavity, synovial hyperplasia and its infiltration with inflammatory cells, as well as cartilage destruction and minimal bone erosion were apparent (Figure 3B). There were only mild inflammatory changes, such as synovial swelling and inflammatory cell influx on the contralateral side showing systemic manifestations of the disease (picture not shown). Histopathological alterations typically seen in arthritic joints of the WT mice were not altered in Tac1^−/−^ and Tacr1^−/−^ mice (Figures 3C, D), while they were reduced in the Tac4^−/−^ and Tac1^−/−/^Tac4^−/−^ groups: synovial swelling, lymphocyte accumulation and cartilage erosion were diminished and signs of bone destruction were not detectable (Figures 3E, F). The semiquantitative scoring obtained from measuring the characteristic inflammatory parameters demonstrates the significantly decreased severity of arthritis in Tac4^−/−^ and Tac1^−/−/^Tac4^−/−^ mice (Figure 3G).

### Role of Tachykinins in Adjuvant-induced IL-1β Production in the Joints

**Figure 4:**
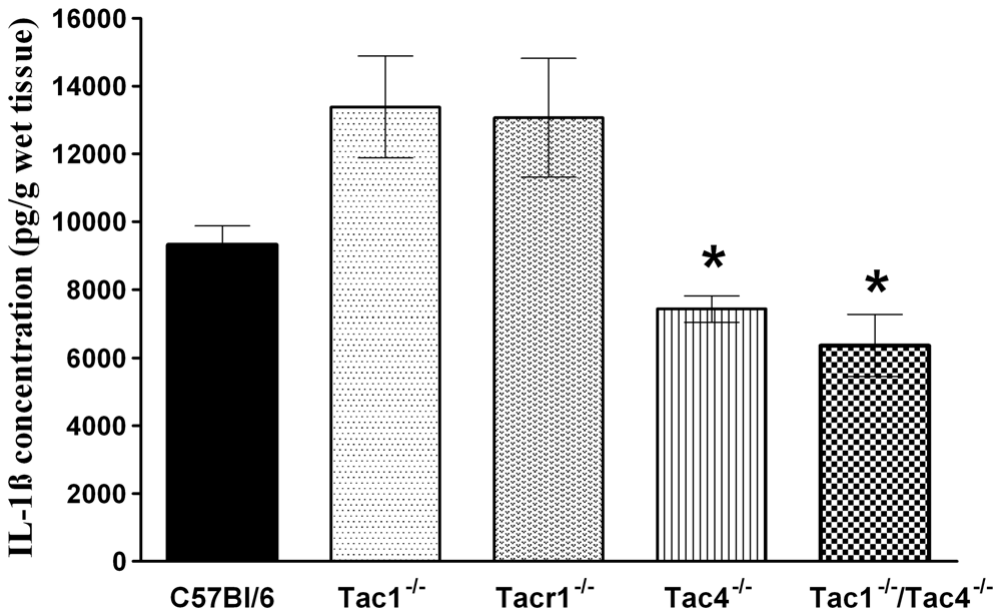
Concentrations of the inflammatory cytokine interleukin-1β (IL-1β) in the joint homogenates on day 21. Each bar represents the mean ± SEM of each group (n = 5–12 mice per group, *p<0.05 vs. C57Bl/6; Kruskal-Wallis followed by Dunn’s post test).

The IL-1β concentration in the intact tibiotarsal joint homogenates of all mouse groups was below the detection limit of the ELISA technique. Adjuvant administration induced an approximately 9000 pg/g production of this inflammatory cytokine in WT control mice. In accordance with the histopathological scoring, IL-1β production was significantly lower in the joints of Tac4^−/−^ and Tac1^−/−/^Tac4^−/−^ double knockout mice compared to WT controls (Figure 4).

## Discussion

The present study provides the first evidence that HK-1 increases inflammatory pain in the chronic phase of CFA-induced arthritis. Although it does not perfectly mimic all pathophysiological processes of rheumatoid arthritis, but is a widely used, well-defined and internationally accepted model with several similarities in the mechanisms (T cell dominant immune response with increase of TNFα, IL-1β, IFNγ, IL-6, IL-17 levels) and symptoms (synovial hyperplasia, inflammatory cell accumulation, cartilage destruction and bone erosion) of the human disease. In addition in several cases results of this model were reliable indicators of efficacy and toxicity of new therapeutics [35], [36], [37]. HK-1 also plays a predominant role in the development of inflammatory morphological alterations and inflammatory cytokine production in the joint. Interestingly, our data reveals for the first time that HK-1 exerts different effects through different mechanisms. While the development of mechanical hyperalgesia involves NK1 receptors, the development of arthritic histopathological changes are not NK1 receptor mediated (Table 1).

**Table 1:** Summary of functional, morphological and immunological alterations in gene-deleted mouse strains compared to the C57Bl/6 WT group.

Mouse strain	Hyperalgesia	Oedema formation	Histopathological alterations	IL-1β
Tac1^−/−^	–	–	–	–
Tacr1^−/−^	↓	–	–	–
Tac4^−/−^	↓	–	↓	↓
Tac1^−/−/^Tac4^−/−^	–	–	↓	↓

Mechanical hyperalgesia was significantly attenuated in Tac4^−/−^ and Tacr1^−/−^ animals, while oedema formation did not differ in any strain. Inflammatory reactions characterised by the histological alterations and changes of cytokine levels were significantly reduced in Tac4^−/−^ and Tac1^−/−/^Tac4^−/−^ groups.

Joints are innervated by capsaicin-sensitive afferents that are not only responsible for nociception and pain sensation, but also exert local and systemic effector functions through the released sensory neuropeptides [4], [5]. Neurogenic inflammatory component defined as vasodilatation, plasma protein extravasation and inflammatory cell recruitment in response to the activation of sensory fibres plays a significant role in rheumatoid arthritis [38]. Transection of sensory nerves reduces hyperalgesia, swelling and joint destruction in artritis models [2]. Our previous results with Transient Receptor Potential Vanilloid 1 (TRPV1) capsaicin receptor deficient mice revealed that the activation of this ion channel by bradykinin, lipoxygenase products and prostanoids enhances the adjuvant-induced oedema, mechanical hyperalgesia and inflammatory reaction in this murine model of arthritis [32]. The release of pro-inflammatory neuropeptides, such as tachykinins (e.g. SP, NKA and NKB), as well as CGRP from these fibers results in neurogenic inflammation around the site of activation. CGRP induces local vasodilatation and SP evokes plasma protein extravasation through NK1 receptor activation on vascular endothelial cells, modulate inflammatory and immune cell functions [4], [5], as well as its central release in the spinal dorsal horn activates the pain pathway [27], [39]. Furthermore, there is a significant number of SP-positive sensory fibres in the synovial tissue. In a healthy joint both the cell lining layer and some nerves branching towards the synovial space are SP-containing [27], [40]. The density of SP-positive sensory fibres is increased in the synovium of RA patients, but decreased in osteoarthritis [41].

Although the patterns and levels of expression of Tac1- and Tac4-derived peptides at early time points prior to the immune response initiation is not known, the present results outline the complexity of the tachykinin system in different mechanisms in arthritis and arthritis-related pain [10], [11]. For decades, SP was the only tachykinin known to be detected by anti-SP antibodies, and a positive readout in a SP radioimmunassay was interpreted as SP immunoreactivity. However the discovery of Tac4 gene-derived peptides changed the interpretation of this paradigm. Since hemokinins and endokinins exhibit structural homology, this consequently results in immunological crossreactivity with anti-SP antibodies. Thus, to date SP and HK-1 cannot be differentiated by radioimmunoassay [16]. It has therefore been suggested, that in several experimental layouts the measured SP-like immunoreactivity reflects both SP and HK-1 contents. Hemokinins were first classified as tachykinins derived from hematopoietic cells [12], but recently they have been shown in immune cells e.g. T and B lymphocytes, macrophages and dendritic cells in the periphery, mediating a broad range of hematopoietic and inflammatory actions [12], [22], [23], [42]. A recent paper has reported that SP and HK-1 are similarly involved in the differentiation of memory CD4+ T cells into Th17 cells via NK1 receptor activation and production of certain cytokines, indicating that SP and HK-1 may act locally on memory T cells to amplify inflammatory responses and could be critical targets in several inflammatory disorders. The other members of the tachykinin family, neurokinins A and B, have no effect on the differentiation of naive and memory T cells [43]. However, HK-1 is also expressed in the sensory nervous system and has a remarkable selectivity and potency for the NK1 receptors [13]. These characteristics allow HK-1 to participate in pain mediation. Nevertheless, HK-1 might have different binding sites on the NK1 receptors, distinct receptor activation mechanisms and signal transduction pathways compared to SP [44]. Furthermore, Endo and colleagues have raised the possibility of a presently unidentified proper receptor related to HK-1 on the basis of several actions of hemokinins different from that of SP [18].

Besides, similarly to SP, HK-1 was also found to enhance the induction of scratching behavior by resiniferatoxin, a TRPV1 agonist, indicating that both HK-1 and SP modulate the response to TRPV1 receptor activation [45].

Concerning chronic autoimmune/inflammatory diseases in human, a recent publication has suggested that HK-1 may be involved in the pathophysiology of inflammatory bowel diseases, such as ulcerative colitis [46], but no data are available on its role in arthritic diseases. However, promising approaches highlight the importance of B cell targeting in arthritis therapy [47]. Since B cells are an important source of HK-1, decreased HK-1 production and release might be an explanation for the efficacy of rituximab, the anti-CD20 monoclonal anibody inducing B cell depletion in rheumatoid arthritis patients [48].

In contrast, the role of NK1 receptors in inflammatory joint diseases has been investigated for decades and a lot of information is available for various disease models and clinical trials. NK1 receptor mRNA is highly expressed in the synovia of RA patients, which is downregulated by tumor necrosis factor alpha [49]. Intraarticular pretreatment with of the NK1 receptor antagonist L-703,606, reduces carrageenan-evoked inflammatory pain, but not oedema in the rat knee [50]. Intaarticular injection of another NK1 receptor antagonist (RP67580) improved the efficacy of dexamethasone to attenuate knee oedema and arthritic allodynia during the experimental period of 7 days of CFA-induced arthritis [51]. Both intraarticular and intraplantar injection of NK1 receptor antagonists 2 days after arthritis induction, reduced pain, oedema and progressive joint destruction in the rat [52].

Adjuvant-induced inflammatory mechanical hyperalgesia, was significantly and similarly reduced from the 11th day of the experiment in Tac4^−/−^ and Tacr1^−/−^ animals, but not in the other knockouts compared to wildtypes suggesting that HK-1 induces hyperalgesia through NK1 receptor activation on sensory neurons. Besides peripheral mechanisms at the nerve terminals, central sensitization in the spinal cord also plays a predominant role in this process.

Although we do not have a precise explanation for why the hyperalgesic action of HK-1 presumably at the NK1 receptors is not observed when SP and NKA are also missing from the system, some hypothesis can be made: a) HK-1 exerts its hyperalgesic effect in the nociceptive pathway through the NK1 tachykinin receptor, but it is counteracted by NKA acting at NK2 receptors in the central nervous system [53], b) HK-1 and SP act at the same receptors (NK1), but they might have different binding sites, affinities and intinsinc efficacies, as well as distinct activation mechanisms and signalling pathways. When both are removed from the system, the inhibition observed in case of the HK-1 absence, might be counteracted via intracellular molecular mechanisms.

There are contradictory data on the role of the tachykinin system in joint swelling. Only some, but not all NK1 receptor antagonists attenuate oedema [50], [51]. Our data also suggest that tachykinins and NK1 receptors are not involved in aduvant-induced swelling.

The inflammatory changes in the joint were also significantly decreased in Tac4 gene-deleted mice, but in contrast to hyperalgesia there were no changes in Tacr1 knockouts. Therefore, a different mechanism seems to mediate the inflammatory functions of HK-1, and a role for a putative HK receptor can be proposed. There are data showing interactions between the tachykinin system and cytokines in rheumatoid arthritis. Monocytes of RA patients secrete greater amounts of TNFα after SP-treatment, compared to monocytes of healthy controls [54]. Reduced substance P release and disease severity were observed after the TNFα inhibitor etanercept treatment in RA patients [55]. Although RA is known as a TNFα dominant disease, other interleukines are also involved in chronic joint destruction. IL-1β plays a role in the immune response modulation and osteoclast activation [56]. In agreement with the histopathological findings, IL-1β decrease in joint homogenates of Tac4 and double gene-deleted mice also suggest that HK-1 acts at another receptor, not the NK1.

In summary, we provided the first evidence for inflammatory and nociceptive roles of HK-1 in a mouse model of chronic arthritis. However, the mechanisms of these actions are different: the peripheral inflammatory effects are not NK1 receptor-mediated, but mechanical hyperalgesia involving central sensitization is dependent on NK1 activation. Based on the present results, identification of the target and the precise signalling pathways, then antagonizing the actions of HK-1 might be a new perspective for the treatment of arthritis.
